# The Causes of Dyspepsia

**Published:** 1891-04

**Authors:** 


### The Causes of Dyspepsia.

One of the most frequent causes of dyspepsia (says Dr. Wm. Pepper), is the constant use of irritating substances, such as tobacco, alcohol and highly seasoned food. Tobacco and strong tea and coffee act both by depressing the nervous force of the stomach and, if swallowed, by directly interfering with the digestive processes. It will not be disputed by any fair-minded person that tobacco, tea and coffee are injurious when taken in excess. It must be admitted that the majority of men, in a state of health, can use a certain amount of tobacco without injury. This amount varies with the individual but is in any case small. I cannot speak too strongly against the filthy and disgusting habit of chewing tobacco.

The *British Journal of Dermatology* gives the following formula for perspiring hands.

R. Eau de Cologne.................. 120 parts.
Tinct. Belladonna............... 15 "

